# Ceftriaxone and the Risk of Ventricular Arrhythmia, Cardiac Arrest, and Death Among Patients Receiving Lansoprazole

**DOI:** 10.1001/jamanetworkopen.2023.39893

**Published:** 2023-10-26

**Authors:** Anthony D. Bai, Amelia Wilkinson, Aws Almufleh, Mandip Rai, Fahad Razak, Amol A. Verma, Siddhartha Srivastava

**Affiliations:** 1 Division of Infectious Diseases, Department of Medicine, Queen’s University, Kingston, Ontario, Canada; 2 Department of Health Research Methods, Evidence and Impact, Faculty of Health Sciences, McMaster University, Hamilton, Ontario, Canada; 3 Division of General Internal Medicine, Department of Medicine, Queen’s University, Kingston, Ontario, Canada; 4 Division of Cardiology, Department of Medicine, Queen’s University, Kingston, Ontario, Canada; 5 Division of Gastroenterology, Department of Medicine, Queen’s University, Kingston, Ontario, Canada; 6 Department of Medicine, University of Toronto, Toronto, Ontario, Canada; 7 Li Ka Shing Knowledge Institute, St Michael’s Hospital, Unity Health Toronto, Toronto, Ontario, Canada; 8 Institute of Health Policy, Management and Evaluation, University of Toronto, Toronto, Ontario, Canada

## Abstract

**Importance:**

The combination of ceftriaxone and lansoprazole has been shown to prolong the corrected QT interval on electrocardiogram. However, it is unknown whether this translates to clinically important patient outcomes.

**Objective:**

To compare lansoprazole with another proton pump inhibitor (PPI) during ceftriaxone treatment in terms of risk for ventricular arrhythmia, cardiac arrest, and in-hospital mortality.

**Design, Setting, and Participants:**

A retrospective cohort study including adult medical inpatients receiving ceftriaxone with lansoprazole or another PPI in 13 hospitals in Ontario, Canada, was conducted from January 1, 2015, to December 31, 2021.

**Exposure:**

Lansoprazole during ceftriaxone treatment vs other PPIs during ceftriaxone treatment.

**Main Outcomes and Measures:**

The primary outcome was a composite of ventricular arrhythmia or cardiac arrest that occurred after hospital admission. The secondary outcome was all-cause in-hospital mortality. Propensity-score weighting was used to adjust for covariates including hospital site, demographic characteristics, comorbidities, risk factors for ventricular arrhythmia, illness severity, admitting diagnoses, and concomitant medications.

**Results:**

Of the 31 152 patients hospitalized on internal medicine wards who were treated with ceftriaxone while receiving a PPI, 16 135 patients (51.8%) were male, and the mean (SD) age was 71.7 (16.0) years. The study included 3747 patients in the lansoprazole group and 27 405 patients in the other PPI group. Ventricular arrhythmia or cardiac arrest occurred in 126 patients (3.4%) within the lansoprazole group and 319 patients (1.2%) within the other PPI group. In-hospital mortality occurred in 746 patients (19.9%) within the lansoprazole group and 2762 patients (10.1%) in the other PPI group. After weighting using propensity scores, the adjusted risk difference for the lansoprazole group minus other PPI group was 1.7% (95% CI, 1.1%-2.3%) for ventricular arrhythmia or cardiac arrest and 7.4% (95% CI, 6.1%-8.8%) for in-hospital mortality.

**Conclusions and Relevance:**

The findings of this cohort study suggest that combination therapy with lansoprazole and ceftriaxone should be avoided. More studies are needed to determine whether these findings could be replicated in other populations and settings.

## Key Points

**Question:**

In adult medical inpatients receiving ceftriaxone treatment, is concomitant lansoprazole compared with other proton pump inhibitors associated with increased risk of ventricular arrhythmia or cardiac arrest and death?

**Findings:**

In a cohort study of 31 152 patients receiving ceftriaxone therapy, concomitant lansoprazole was associated with an adjusted absolute risk increase of 1.7% for ventricular arrhythmia or cardiac arrest compared with other proton pump inhibitors. The risk was greater, at 7.4%, for in-hospital mortality.

**Meaning:**

The findings of this study suggest that avoiding the combination of ceftriaxone and lansoprazole may decrease the risk of ventricular arrhythmia, cardiac arrest, and mortality.

## Teaser

This cohort study compares the risk of ventricular arrhythmia, cardiac arrest, and in-hospital mortality with concomitant use of ceftriaxone with lansoprazole vs ceftriaxone with other proton pump inhibitors among adult medical inpatients.

## Introduction

Ceftriaxone and lansoprazole are commonly prescribed medications.^1,2^ In a single-center retrospective cohort study of 380 000 patients, lansoprazole combined with ceftriaxone was associated with prolonged corrected QT (QTc) intervals.^3^ On average, QTc intervals were 12 milliseconds (95% CI, 7-15 milliseconds) longer in men and 9 milliseconds (95% CI, 5.2-11.3 milliseconds) longer in women who were receiving ceftriaxone and lansoprazole concurrently compared with patients receiving either drug alone.^3^ This interaction with ceftriaxone was not observed with other proton pump inhibitors (PPIs).^3^ As a potential mechanism of action, ceftriaxone and lansoprazole were shown to block the hERG potassium channel in patch-clamp electrophysiologic experiments.^3^ The exact molecular mechanism remains unclear.^3^ It is unknown whether prolonged QTc intervals translates to important outcomes for patients, including ventricular arrhythmia, cardiac arrest, or death.

Combination therapy with ceftriaxone and lansoprazole is common in patients admitted to the internal medicine ward in hospitals. These patients often have multiple comorbidities and medications requiring PPI treatment or prophylaxis. Furthermore, there is a high prevalence of inappropriate PPI use in this population of older adults.^4^ At the same time, patients in internal medicine wards are often admitted with pneumonia, urinary tract infection, or sepsis that is treated with ceftriaxone.^1^ We conducted a retrospective cohort study to answer the following question: in adult medical inpatients receiving ceftriaxone treatment, is concomitant lansoprazole compared with other PPIs associated with increased risk of ventricular arrhythmia, cardiac arrest, and death in the hospital?

## Methods

We conducted a multicenter, retrospective cohort study across 13 hospitals in Ontario, Canada. The Unity Health Toronto Research Ethics Board approved this study with waiver of informed consent because deidentified data were collected retrospectively. The study is reported as per the Strengthening the Reporting of Observational Studies in Epidemiology (STROBE) reporting guideline.

### Patient Population

The study included consecutive adult patients admitted to a medical inpatient service at the 13 hospitals from January 1, 2015, to December 31, 2021, who fulfilled 2 criteria. First, the patient was prescribed 1 or more doses of parenteral ceftriaxone during their hospital stay. Second, the patient was prescribed a PPI during the period between the first and last dose of ceftriaxone. Patients who were not receiving any PPI during ceftriaxone treatment were excluded. This was a convenient sample size based on the study date cutoff.

### Data Source

This study used data from the GEMINI database of internal medicine patients admitted to participating hospitals in Ontario, Canada.^5^ The GEMINI database contains patient-level linked administrative and clinical data on hospitalization. Administrative data included hospital data as reported to the Canadian Institute for Health Information Discharge Abstract Database and National Ambulatory Care Reporting System, which contain demographic characteristics, diagnoses, interventions, resource use, and disposition during hospital stay and emergency department visit.^5^ Diagnoses prior to, on, and after hospital admission were coded as per the enhanced Canadian version of the *International Statistical Classification of Diseases and Related Health Problems, 10th Revision* (*ICD-10-CA*).^5^ In addition, the GEMINI database includes data from hospital electronic information systems that contain medication orders captured in pharmacy information systems and bloodwork results while the patient is hospitalized.^5^ Data sources and linkage are described in detail elsewhere.^5,6^

### Exposure

The exposure of interest was concomitant lansoprazole during the period between the first and last dose of ceftriaxone. The comparison group was composed of patients who were prescribed a PPI other than lansoprazole during the period between the first and last doses of ceftriaxone. Other PPIs included pantoprazole, rabeprazole, esomeprazole, and omeprazole by any route. Patients who were prescribed lansoprazole in addition to another PPI during ceftriaxone therapy were excluded from the comparison group and included within the lansoprazole group.

### Outcomes

Patients were followed up until hospital discharge. The primary outcome was a composite of ventricular arrhythmia or cardiac arrest that developed during hospital stay and postadmission based on *ICD-10-CA* codes (eTable 1 in Supplement 1). This definition excluded ventricular premature depolarization, other premature depolarization, and unspecified cardiac arrhythmias. The exact date for the occurrence of the primary outcome was not recorded, so time-to-event analysis was not possible. The secondary outcome was all-cause in-hospital mortality.

### Covariates

The following covariates were collected because they were considered significant factors: (1) demographic characteristics (age, sex, resident of a long-term care home; data on race and ethnicity are not captured in the database), (2) hospital admission (hospital site, admission year, admission meteorologic season), (3) comorbidities before admission (modified Charlson comorbidity index^7^ that excluded myocardial infarction, heart failure, and chronic kidney disease, because these comorbidities were considered as individual risk factors), (4) admitting diagnosis (10 most common admitting diagnoses for the study cohort), (5) illness severity (modified Laboratory-Based Acute Physiology Score within 24 hours of admission^8^ based on laboratory parameters in which a higher score correlates with higher in-hospital mortality,^9,10^ intensive care unit [ICU] admission before the first dose of ceftriaxone and PPI), (6) risk factors for ventricular arrhythmia (eTable 1 in Supplement 1) (coronary artery disease, prior myocardial infarction, heart failure with preserved or reduced ejection fraction, cardiomyopathy, history of ventricular arrhythmia or cardiac arrest, chronic kidney disease, abnormal serum potassium level (<3.5 or >5 mEq/L [to convert to millimoles per liter, multiply by 1] within 24 hours of admission), and (7) systemic medication classes prescribed before the first dose of ceftriaxone that may increase the risk of ventricular arrhythmia based on the American Heart Association scientific statement (fluoroquinolones, macrolides, cardiac medications, other medications).^11^ eTable 2 in Supplement 1 provides the full list of medications.

### Statistical Analysis

Complete case analysis was done in which patients with any missing data were excluded, because the proportion of missing data was less than 5%.^12,13^ Descriptive statistics included mean (SD) for normally distributed continuous variables, median (IQR) for nonnormally distributed continuous variables, and counts with percentages for categorical variables. The balance of covariates between the 2 groups is described using absolute standardized mean difference (ASMD).

Primary and secondary outcomes were compared between the lansoprazole group and other PPI group using the χ^2^ test. A risk difference was calculated as risk in the lansoprazole group minus risk in the other PPI group with a CI estimated using the Miettinen-Nurminen method.^14^ In addition, a risk ratio was calculated with a CI estimated using the normal approximation method. To address potential bias, the propensity score for lansoprazole was estimated using a logistic regression of all the aforementioned covariates. Covariates were balanced using overlap weights.^15^ Overlap weighting of propensity scores between 2 groups based on logistic regression model will always lead to an exact balance in the means of any included covariate,^15,16^ so the ASMD will be 0. The weighted difference in means for the outcomes would be the average risk difference, and its SEs were used to estimate the CI.^15,17^ Risk ratio and CI were also calculated after overlap weighting to derive the E value, which is the minimum risk ratio that an unmeasured confounder would need to have with treatment and outcome to fully explain the treatment-outcome association.^18^

In a subgroup analysis, patients who were admitted to the ICU before their first dose of ceftriaxone and PPI were excluded. This was based on the rationale that ICU admission at baseline could be a confounder, because patients in ICUs were more likely to receive PPI therapy for prophylaxis postintubation or treatment of gastrointestinal bleed. At the same time, patients in ICUs had a higher risk of ventricular arrhythmia, cardiac arrest, and death due to their critical illness. In this subgroup analysis, we added an exploratory outcome of ICU transfer after the first dose of ceftriaxone and PPI, which could be an intermediate factor that followed ventricular arrhythmia or cardiac arrest and preceded death.

Patients may have taken more than one type of PPI during their hospital stay. To address this potential contamination bias, we performed a subgroup analysis that compared patients who received only lansoprazole with patients who received only pantoprazole without being exposed to any other PPI. Pantoprazole was chosen because it had the largest number of patients.

Four sensitivity analyses were added post hoc: (1) analysis that included patients with missing data and assumed the best worst-case scenario for unknown exposure; (2) analysis that accounted for ceftriaxone and PPI duration in the propensity score; (3) subgroup analysis of patients who began receiving ceftriaxone and a PPI within 1 day of admission, which would result in the primary outcome of ventricular arrhythmia or cardiac arrest occurring after exposure; and (4) subgroup analysis that excluded pantoprazole, because it could be given intravenously for gastrointestinal bleeding. Thus, this subgroup analysis compared only oral PPIs.

Reported CIs were 2-sided 95% intervals, and all tests were 2-sided with a *P* < .05 significance level. The statistical software R, version 4.1.3 (R Foundation for Statistical Computing) was used for analysis. The statistical package PSweight was used for propensity score weighting.^17^

## Results

### Baseline Characteristics

Of the 31 152 patients who were admitted to the internal medicine service and treated with ceftriaxone while receiving a PPI, 16 135 patients (51.8%) were men, 15 017 (48.2%) were women, and the mean (SD) age was 71.7 (16.0) years. Timing and duration of ceftriaxone and PPI are described in eTable 3 in Supplement 1. Follow-up was complete for all patients. The proportion of patients with missing data who were excluded from the study was 3.5% (Figure).

**Figure.**
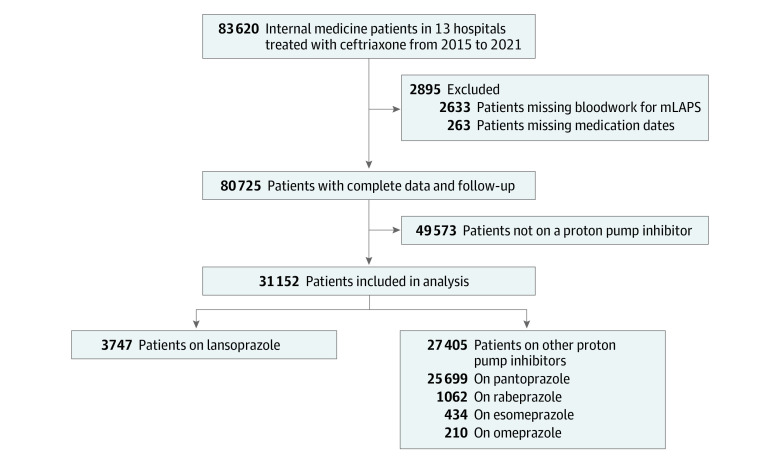
Flow Diagram One patient had both missing bloodwork for modified Laboratory-Based Acute Physiology Score (mLAPS) and missing medication dates.

There were 3747 patients (12.0%) in the lansoprazole group and 27 405 patients (88.0%) in the other PPI group (Figure). The excluded patients who were not prescribed any PPI and patients with missing data are described in eTable 4 in Supplement 1. The proportion of patients who were also prescribed another PPI during hospital stay are described in eTable 5 in Supplement 1.

Baseline characteristics of the lansoprazole group and other PPI group are described in Table 1. Patients in the lansoprazole group were more likely to be older, reside in a long-term care home, admitted during 2020, given a higher modified Laboratory-Based Acute Physiology Score, admitted to the ICU, admitted for aspiration or COVID-19, and receiving medications associated with ventricular arrhythmia (Table 1). The distribution of PPI prescriptions across the 13 hospital sites is presented in eTable 6 in Supplement 1.

**Table 1.** Baseline Characteristics

Characteristic	No. (%)		ASMD
	Lansoprazole (n = 3747)	Other PPI (n = 27 405)	
Age, mean (SD), y	74.0 (15.9)	71.4 (16.0)	0.162
Sex			
Female	1772 (47.3)	13 245 (48.3)	0.021
Male	1975 (52.7)	14 160 (51.7)	0.021
From a long-term care home	574 (15.3)	1595 (5.8)	0.313
Hospital sites			
A	452 (12.1)	5118 (18.7)	0.184
B	0	10 (0.04)	0.027
C	439 (11.7)	3206 (11.7)	0.001
D	431 (11.5)	1246 (4.6)	0.258
E	48 (1.3)	415 (1.5)	0.020
F	223 (6.0)	693 (2.5)	0.170
G	8 (0.2)	890 (3.3)	0.234
H	116 (3.1)	2325 (8.5)	0.232
I	43 (1.2)	1135 (4.1)	0.187
J	562 (15.0)	2601 (9.5)	0.169
K	760 (20.3)	2818 (10.3)	0.281
L	338 (9.0)	4168 (15.2)	0.191
M	327 (8.7)	2780 (10.1)	0.048
Admission year			
2015	246 (6.6)	2427 (8.9)	0.086
2016	390 (10.4)	3312 (12.1)	0.053
2017	548 (14.6)	4108 (15.0)	0.010
2018	655 (17.5)	4331 (15.8)	0.045
2019	573 (15.3)	4613 (16.8)	0.042
2020	923 (24.6)	5391 (19.7)	0.120
2021	412 (11.0)	3223 (11.8)	0.024
Admission season			
Winter	974 (26.0)	6887 (25.1)	0.020
Spring	996 (26.6)	6837 (25.0)	0.037
Summer	901 (24.1)	7005 (25.6)	0.035
Autumn	876 (23.4)	6676 (24.4)	0.023
Modified Charlson comorbidity index, mean (SD)	1.3 (1.7)	1.4 (1.9)	0.068
Risk factors for ventricular arrhythmia			
Coronary artery disease	94 (2.5)	728 (2.7)	0.009
Prior myocardial infarction	68 (1.8)	551 (2.0)	0.014
Cardiomyopathy	43 (1.2)	251 (0.9)	0.023
Heart failure	484 (12.9)	3407 (12.4)	0.015
Prior ventricular arrhythmia	7 (0.2)	69 (0.3)	0.014
Chronic kidney disease	203 (5.4)	1748 (6.4)	0.041
Abnormal serum potassium level at admission	1706 (45.5)	11 614 (42.4)	0.064
mLAPS score, mean (SD)	27.0 (18.5)	24.5 (16.8)	0.140
ICU admission prior to ceftriaxone and PPI	349 (9.3)	509 (1.9)	0.329
Admitting main responsible diagnosis			
Pneumonia	251 (6.7)	1881 (6.9)	0.007
Urinary tract infection	203 (5.4)	1642 (6.0)	0.025
Aspiration	418 (11.2)	718 (2.6)	0.342
COPD with lower respiratory tract infection	109 (2.9)	1166 (4.3)	0.072
Congestive heart failure	103 (2.8)	1030 (3.8)	0.057
COPD exacerbation	47 (1.3)	874 (3.2)	0.132
Sepsis	107 (2.9)	555 (2.0)	0.054
COVID-19 infection	144 (3.8)	448 (1.6)	0.136
Cellulitis involving limb	15 (0.4)	293 (1.1)	0.078
Acute kidney failure	53 (1.4)	326 (1.6)	0.012
Medications taken during ceftriaxone therapy			
Quinolones	162 (4.3)	796 (2.9)	0.076
Macrolides	109 (2.9)	536 (2.0)	0.062
Cardiac medications associated with ventricular arrhythmia	226 (6.0)	615 (2.2)	0.191
Other medications associated with ventricular arrhythmia	704 (18.8)	3532 (12.9)	0.162

Abbreviations: ASMD, absolute standardized mean difference; COPD, chronic obstructive pulmonary disease; ICU, intensive care unit; mLAPS, modified Laboratory-Based Acute Physiology Score; PPI, proton pump inhibitor.

### Outcomes

There were 445 patients who had ventricular arrhythmia or cardiac arrest; of these, 336 patients (75.5%) died in the hospital. Ventricular arrhythmia or cardiac arrest occurred in 126 patients (3.4%) from the lansoprazole group and 319 patients (1.2%) from the other PPI group (*P* < .001), with an unadjusted risk difference of 2.2% (95% CI, 1.7%-2.8%) (Table 2). The association between cardiac risk factors and ventricular arrhythmia or cardiac arrest is reported in eTable 7 in Supplement 1. All-cause in-hospital mortality occurred in 746 patients (19.9%) in the lansoprazole group and 2762 patients (10.1%) in the other PPI group (*P* < .001) with an unadjusted risk difference of 9.8% (95% CI, 8.5%-11.2%). The risk for the outcomes for each calendar year remained similar from 2015 to 2021 (eTable 8 in Supplement 1). The median length of stay was 12.6 (IQR, 6.1-28.4) days in the lansoprazole group and 7.0 (IQR, 3.8-13.7) days in the other PPI group.

**Table 2.** Outcomes During Hospital Stay

Outcome	No. (%)		Risk ratio (95% CI)		E-value (95% CI)^a^	Risk difference in % (95% CI)	
	Lansoprazole (n = 3747)	Other PPI (n = 27 405)	Unadjusted	Adjusted		Unadjusted	Adjusted
Ventricular arrhythmia or cardiac arrest	126 (3.4)	319 (1.2)	2.9 (2.4-3.5)	2.2 (1.7-2.7)^b^	3.7 (2.8-4.9)	2.2 (1.7-2.8)	1.7 (1.1-2.3)^b^
Ventricular arrhythmia	8 (0.2)	25 (0.1)	NA	NA	NA	NA	NA
Cardiac arrest	118 (3.2)	294 (1.1)	NA	NA	NA	NA	NA
All cause in-hospital mortality	746 (19.9)	2762 (10.1)	2.0 (1.8-2.1)	1.6 (1.5-1.7)^b^	2.6 (2.3-2.9)	9.8 (8.5-11.2)	7.4 (6.1-8.8)^b^

Abbreviations: NA, not applicable; PPI, proton pump inhibitor.

^a^ E value based on adjusted relative risk.

^b^ Adjusted risk after overlap weighting of propensity scores.

### Propensity Score Adjustment

After adjustment by overlap weights, the overlap population is described in Table 3. The ASMD was 0 for each covariate after overlap weighting. The lansoprazole group still had a significantly higher risk of ventricular arrhythmia or cardiac arrest and all-cause in-hospital mortality (Table 2). The lansoprazole group had an adjusted risk difference of 1.7% (95% CI, 1.1%-2.3%) for ventricular arrhythmia or cardiac arrest, which corresponded to a number needed to harm of 58.8 (95% CI, 43.5-90.9). The adjusted risk ratio was 2.2 (95% CI, 1.7-2.2) and E value was 3.7 (95% CI, 2.8-4.9). For mortality, the adjusted risk difference was 7.4% (95% CI, 6.1%-8.8%). The adjusted risk ratio was 1.6 (95% CI, 1.5-1.7) and the E value was 2.6 (95% CI, 2.3-2.9).

**Table 3.** Balance of Factors for the Overlap Population After Propensity Score Weighting

Factor	%	
	Lansoprazole effective sample size (n = 3586.4)	Other PPI effective sample size (n = 15 425.2)
Age, mean (SD), y	73.6 (16.1)	73.6 (15.8)
Sex		
Female	48.1	48.1
Male	51.9	51.9
From a long-term care home	12.6	12.6
Hospital sites		
A	13.3	13.3
B	0	0
C	12.2	12.2
D	10.4	10.4
E	1.4	1.4
F	5.3	5.3
G	0.3	0.3
H	3.5	3.5
I	1.4	1.4
J	14.5	14.5
K	18.9	18.9
L	9.9	9.9
M	9.0	9.0
Admission year		
2015	6.7	6.7
2016	10.5	10.5
2017	14.9	14.9
2018	17.3	17.3
2019	15.4	15.4
2020	24.0	24.0
2021	11.3	11.3
Admission season		
Winter	26.0	26.0
Spring	26.2	26.2
Summer	24.5	24.5
Autumn	23.3	23.3
Modified Charlson comorbidity index, mean (SD)	1.3 (1.7)	1.3 (1.8)
Risk factors for ventricular arrhythmia		
Coronary artery disease	2.5	2.5
Prior myocardial infarction	1.9	1.9
Cardiomyopathy	1.1	1.1
Heart failure	12.8	12.8
Prior ventricular arrhythmia	0.2	0.2
Chronic kidney disease	5.6	5.6
Abnormal serum potassium level at admission	45.1	45.1
mLAPS score, mean (SD)	26.5 (18.3)	26.5 (18.1)
ICU admission prior to ceftriaxone and PPI	6.2	6.2
Admitting main responsible diagnosis		
Pneumonia	6.9	6.9
Urinary tract infection	5.7	5.7
Aspiration	8.0	8.0
COPD with lower respiratory tract infection	3.2	3.2
Congestive heart failure	3.0	3.0
COPD exacerbation	1.5	1.5
Sepsis	2.9	2.9
COVID-19 infection	3.0	3.0
Cellulitis involving limb	0.5	0.5
Acute kidney failure	1.5	1.5
Medications taken during ceftriaxone therapy		
Quinolones	3.8	3.8
Macrolides	2.5	2.5
Cardiac medications associated with ventricular arrhythmia	4.5	4.5
Other medications associated with ventricular arrhythmia	17.1	17.1

Abbreviations: COPD, chronic obstructive pulmonary disease; ICU, intensive care unit; mLAPS, modified Laboratory-Based Acute Physiology Score; PPI, proton pump inhibitor.

### Subgroup and Sensitivity Analyses

Subgroup and sensitivity analyses yielded similar result of a higher risk of ventricular arrhythmia or cardiac arrest and mortality in the lansoprazole group. The results are presented in eTables 9-14 in Supplement 1.

## Discussion

This large, multicenter, retrospective cohort study compared 3747 patients receiving lansoprazole during ceftriaxone therapy vs 27 405 patients receiving other PPIs during ceftriaxone therapy. The lansoprazole group had higher risk of ventricular arrhythmia or cardiac arrest with an adjusted risk difference of 1.7% (95% CI, 1.1%-2.3%). This corresponded to a number needed to harm of 58.8. In addition, the lansoprazole group had a higher risk of all-cause in-hospital mortality with an adjusted risk difference of 7.4% (95% CI, 6.1%-8.8%).

To our knowledge, this is the first study that examined the interaction between ceftriaxone and lansoprazole using important patient outcomes, including ventricular arrhythmia, cardiac arrest, and mortality. In a prior study, the absolute risk of a clinically significant QTc interval greater than 500 milliseconds was increased by 4% to 7% in the ceftriaxone and lansoprazole group compared with either drug alone.^3^ One research letter described a patient in whom the QTc interval increased by 58 milliseconds when lansoprazole was added to ceftriaxone and 2 patients who received lansoprazole and ceftriaxone in a prospective cohort of 40 patients with torsade de pointes and sudden cardiac death.^19^ However, no conclusion could be drawn from these anecdotes. Our study furthers the knowledge by observing an increased risk of ventricular arrhythmia, cardiac arrest, and death when ceftriaxone was combined with lansoprazole.

This study has important implications. If ceftriaxone and lansoprazole may lead to serious adverse effects, including ventricular arrhythmia, cardiac arrest, and death, then this combination should be avoided in clinical practice given the existence of safer alternatives. Ventricular arrhythmia and cardiac arrest could be considered potential adverse events when PPIs and antibiotics are inappropriately prescribed. Ceftriaxone and lansoprazole should not be prescribed unless there is a clear indication. For patients receiving lansoprazole who are going to receive ceftriaxone, lansoprazole should either be withheld or substituted by another PPI. This simple medication change has the potential to prevent major patient morbidity and mortality.

### Strengths and Limitations

This study has several strengths. First, we developed the research question a priori based on a prior study that showed the association of ceftriaxone and lansoprazole with a prolonged QTc interval as well as a potential mechanism of action for this interaction.^3^ Second, we had a large sample size of more than 30 000 patients across 13 hospitals, including both academic and community sites, which was adequate to detect a signal between the 2 groups for a relatively rare event of ventricular arrhythmia and cardiac arrest that occurred in 1% to 3% of the patients. This suggests the findings are generalizable. Third, we used an appropriate comparison group of patients receiving other PPIs. As reported in eTable 4 in Supplement 1, compared with patients not receiving a PPI, patients receiving PPIs had more comorbidities and medications, which may predispose them to the outcome of ventricular arrhythmia, cardiac arrest, and death. The comparison of lansoprazole with other PPIs instead of no PPI addresses this confounding by indication, at least in part, because patients receiving lansoprazole and other PPIs were more likely to share similar comorbidities and concomitant medications. Proton pump inhibitors as a class may also prolong the QTc interval.^20,21^ Therefore, the comparison group of other PPIs was also useful to confirm that the drug interaction was specific to lansoprazole and excluded a PPI class effect by itself or with ceftriaxone.

The study has limitations. First, QTc intervals on electrocardiograms were not captured in our study, limiting our ability to definitively link the observed outcomes with QTc interval prolongation. However, prolonged QTc interval is an intermediate measure, which is trivial compared with the important clinical outcomes for patients of ventricular arrhythmia, cardiac arrest, and death.

Second, our study could not confirm the temporal relationship of the ventricular arrhythmia or cardiac arrest event relative to ceftriaxone and lansoprazole treatment, because the study data did not capture the specific date and time for the ventricular arrhythmia or cardiac arrest event. We specified that this outcome must have occurred postadmission and ceftriaxone or PPI therapy was initiated in more than 84% of patients within 1 day of admission, suggesting it was unlikely for the outcome to occur prior to the exposure. Moreover, the sensitivity analysis that included only patients who received ceftriaxone and a PPI within 1 day of admission showed similar results (eTable 14 in Supplement 1).

Third, ICU admission could be both a confounder and consequence of the outcome. To account for this, we adjusted for ICU admission before the first dose of ceftriaxone and PPI as a covariate. The subgroup analysis excluding patients admitted to the ICU before ceftriaxone and PPIs were prescribed showed similar results. In this subgroup analysis, we also included transfer to the ICU after the start of ceftriaxone and PPI therapy as an exploratory outcome. There was no significant difference in ICU transfer between the 2 groups (eTable 10 in Supplement 1). This could be explained by the fact that ICU transfer was not necessarily within the pathway downstream from ventricular arrhythmia or cardiac arrest. Patients were transferred to the ICU after a ventricular arrhythmia or cardiac arrest only if they were designated to receive all resuscitation procedures, had a witnessed event with prompt start of cardiopulmonary resuscitation, and achieved return of spontaneous circulation.

Fourth, propensity score adjustment could only balance measured covariates and there may still be residual confounding. The lansoprazole group had a much higher in-hospital mortality rate than the other PPI group, with a risk difference of 7.4%. This was much higher than expected given that a prior study showed that the ceftriaxone and lansoprazole combination increased the risk of prolonged QTc interval by 4% to 7%^3^ and this combination increased the risk of ventricular arrhythmia or cardiac arrest by 1.7% in our study. A portion of this mortality gap may be due to residual confounding. The E value of 2.6 for in-hospital mortality risk ratio and 3.7 for ventricular arrhythmia and cardiac arrest suggest a robust signal for harm. It would be unlikely for an unmeasured confounder to explain this signal.

## Conclusions

In this cohort study of 31 152 patients, the combination of ceftriaxone and lansoprazole was associated with an increased risk of ventricular arrhythmia, cardiac arrest, and mortality. We acknowledge that newly discovered significant associations are often inflated.^22^ Therefore, we suggest a cautious interpretation of our results. It would be important for future observational studies to try to replicate our findings. Until there is more evidence, findings from our study suggest that when prescribing ceftriaxone for patients receiving lansoprazole, the need for the PPI should be examined and, if indicated, substitution with another PPI may be safer.

## Notes

Supplement 1. **eTable 1.** *ICD-10-CA* Codes for Risk Factors of Ventricular Arrhythmia and Primary Outcome **eTable 2.** List of Medications That Increase Risk of Ventricular Arrhythmia **eTable 3.** Timing and Duration of Ceftriaxone and PPI **eTable 4.** Excluded Patients on No Proton Pump Inhibitor and Patients With Missing Data **eTable 5.** Proportion of Patients Who Were Also Prescribed Another Proton Pump Inhibitor During Hospital Stay in the Lansoprazole and Other Proton Pump Inhibitor Group **eTable 6.** Hospital Sites Description **eTable 7.** The Association of A Priori Selected Predictor With Ventricular Arrhythmia or Cardiac Arrest in Logistic Regression Model **eTable 8.** Risk of Ventricular Arrhythmia or Cardiac Arrest, and In-Hospital Mortality per Year From 2015 to 2021 **eTable 9.** Baseline Characteristics Within Subgroup Analysis of Patients Not Admitted to ICU Prior to Ceftriaxone and PPI **eTable 10.** Outcomes Within Subgroup Analysis of Patients Not Admitted to ICU Prior to Ceftriaxone and PPI **eTable 11.** Baseline Characteristics Within Subgroup Analysis of Patients Who Received Either Only Lansoprazole or Only Pantoprazole Without Being Exposed to Any Other PPI During Hospital Stay **eTable 12.** Outcomes Within Subgroup Analysis of Patients Who Received Either Only Lansoprazole or Only Pantoprazole Without Being Exposed to Any Other PPI During Hospital Stay **eTable 13.** Sensitivity Analysis of Outcomes During Hospital Stay That Includes Patients With Missing mLAPS and Assumes Best-Worst-Case Scenario for Unknown Exposure **eTable 14.** Other Sensitivity Analyses

Supplement 2.
Data Sharing Statement
