# Supplementary material for: Ceftriaxone and the Risk of Ventricular Arrhythmia, Cardiac Arrest, and Death Among Patients Receiving Lansoprazole
Source: JAMA Netw Open. 2023 Oct 26;6(10):e2339893. doi: 10.1001/jamanetworkopen.2023.39893 (PMC10603497; doi:10.1001/jamanetworkopen.2023.39893)
Supplement: Supplement 1. — eTable 1. ICD-10-CA Codes for Risk Factors of Ventricular Arrhythmia and Primary Outcome eTable 2. List of Medications That Increase Risk of Ventricular Arrhythmia eTable 3. Timing and Duration of Ceftriaxone and PPI eTable 4. Excluded Patients on No Proton Pump Inhibitor and Patients With Missing Data eTable 5. Proportion of Patients Who Were Also Prescribed Another Proton Pump Inhibitor During Hospital Stay in the Lansoprazole and Other Proton Pump Inhibitor Group eTable 6. Hospital Sites Description eTable 7. The Association of A Priori Selected Predictor With Ventricular Arrhythmia or Cardiac Arrest in Logistic Regression Model eTable 8. Risk of Ventricular Arrhythmia or Cardiac Arrest, and In-Hospital Mortality per Year From 2015 to 2021 eTable 9. Baseline Characteristics Within Subgroup Analysis of Patients Not Admitted to ICU Prior to Ceftriaxone and PPI eTable 10. Outcomes Within Subgroup Analysis of Patients Not Admitted to ICU Prior to Ceftriaxone and PPI eTable 11. Baseline Characteristics Within Subgroup Analysis of Patients Who Received Either Only Lansoprazole or Only Pantoprazole Without Being Exposed to Any Other PPI During Hospital Stay eTable 12. Outcomes Within Subgroup Analysis of Patients Who Received Either Only Lansoprazole or Only Pantoprazole Without Being Exposed to Any Other PPI During Hospital Stay eTable 13. Sensitivity Analysis of Outcomes During Hospital Stay That Includes Patients With Missing mLAPS and Assumes Best-Worst-Case Scenario for Unknown Exposure eTable 14. Other Sensitivity Analyses [file jamanetwopen-e2339893-s001.pdf]

Supplementary Online Content

Bai AD, Wilkinson A, Almufleh AS, et al. Ceftriaxone and the risk of ventricular arrhythmia, cardiac arrest, and death among patients receiving lansoprazole. *JAMA Netw Open*. 2023;6(10):e2339893. doi:10.1001/jamanetworkopen.2023.39893

eTable 1. *ICD-10-CA* Codes for Risk Factors of Ventricular Arrhythmia and Primary Outcome

eTable 2. List of Medications That Increase Risk of Ventricular Arrhythmia

eTable 3. Timing and Duration of Ceftriaxone and PPI

eTable 4. Excluded Patients on No Proton Pump Inhibitor and Patients With Missing Data

eTable 5. Proportion of Patients Who Were Also Prescribed Another Proton Pump Inhibitor During Hospital Stay in the Lansoprazole and Other Proton Pump Inhibitor Group

eTable 6. Hospital Sites Description

eTable 7. The Association of A Priori Selected Predictor With Ventricular Arrhythmia or Cardiac Arrest in Logistic Regression Model

eTable 8. Risk of Ventricular Arrhythmia or Cardiac Arrest, and In-Hospital Mortality per Year From 2015 to 2021

eTable 9. Baseline Characteristics Within Subgroup Analysis of Patients Not Admitted to ICU Prior to Ceftriaxone and PPI

eTable 10. Outcomes Within Subgroup Analysis of Patients Not Admitted to ICU Prior to Ceftriaxone and PPI

eTable 11. Baseline Characteristics Within Subgroup Analysis of Patients Who Received Either Only Lansoprazole or Only Pantoprazole Without Being Exposed to Any Other PPI During Hospital Stay

eTable 12. Outcomes Within Subgroup Analysis of Patients Who Received Either Only Lansoprazole or Only Pantoprazole Without Being Exposed to Any Other PPI During Hospital Stay

eTable 13. Sensitivity Analysis of Outcomes During Hospital Stay That Includes Patients With Missing mLAPS and Assumes Best-Worst-Case Scenario for Unknown Exposure

eTable 14. Other Sensitivity Analyses

This supplementary material has been provided by the authors to give readers additional information about their work.

© 2023 Bai D et al. *JAMA Network Open.*

eTable 1. *ICD-10-CA* codes for risk factors of ventricular arrhythmia and primary outcome

Diagnoses	*ICD-10-CA* codes
Risk factors for ventricular arrhythmia	
Coronary artery disease	I20.x, I24.x<br>I25.1, I25.6, I25.8, I25.9<br>Z95.1
Prior myocardial infarction	I21.x, I22.x, I23.x<br>I24.1, I25.2
Heart failure	I11.0<br>I13.0, I13.2<br>I50.x<br>I97.1<br>T86.2, T86.3
Cardiomyopathy	I25.5<br>I42.x, I43.x<br>I51.7<br>E63.9<br>O90.3
Prior history of ventricular arrhythmia or cardiac arrest	I46.x<br>I47.0, I47.2<br>I49.0, I49.3, I49.4, I49.8
Chronic kidney disease	I12.0, I13.1<br>N03.x, N05.x, N18.x, N19.x<br>N25.0<br>Z49.x<br>Z94.0, Z99.0
Outcome	
Ventricular arrhythmia	I47.0 (re-entry ventricular arrhythmia)<br>I47.2 (ventricular tachycardia)<br>I49.0x (ventricular fibrillation and flutter)
Cardiac arrest	I46.x (cardiac arrest)

x means all possible numbers
Reference for ICD-10-CA codes: https://secure.cihi.ca/estore/productSeries.htm?pc=PCC189

© 2023 Bai D et al. *JAMA Network Open.*

eTable 2. List of medications that increase risk of ventricular arrhythmia

Category	Medications
Fluoroquinolones	ciprofloxacin, moxifloxacin, levofloxacin
Macrolides	azithromycin, erythromycin, clarithromycin
Cardiac medications	amiodarone, disopyramide, dofetilide, dronedarone, hydroquinidine, flecainide, ibutilide, niferidil, procainamide, propafenone, quinidine, sotalol, digoxin, dobutamine, milrinone, epinephrine, norepinephrine, dopamine, isoprenaline, ephedrine
Other medications	bupropion, escitalopram, citalopram, desipramine, imipramine, trazodone, venlafaxine, lithium, chlorpromazine, thioridazine, haloperidol, levomeprazin, levosulpride, pimozide, ulpiride, sultopride, domperidone, ondansetron, metoclopramide, diphenhydramine, fluconazole, itraconazole, ketoconazole, methadone, donepezil, hydroxychloroquine, chloroquine

Reference: Tisdale JE, Chung MK, Campbell KB, Hammadah M, Joglar JA, Leclerc J, Rajagopalan B, American Heart Association Clinical Pharmacology Committee of the Council on Clinical Cardiology and Council on Cardiovascular and Stroke Nursing. Drug-induced arrhythmias: a scientific statement from the American Heart Association. *Circulation.* 2020;142(15):e214-33.

© 2023 Bai D et al. *JAMA Network Open.*

eTable 3. Timing and duration of ceftriaxone and PPI

	Description
Time from admission to first dose of ceftriaxone	Median (IQR) of 0 (0,1) days<br>26,532 (85.2%) patients received ceftriaxone within 1 day of admission
Duration of ceftriaxone treatment	Median (IQR) of 4 (1,6) days for all patients<br>Median (IQR) of 6 (2, 8) days for the lansoprazole group<br>Median (IQR) of 3 (1, 6) days for the other PPI group
Time from admission to first dose of PPI	Median (IQR) of 0 (0,1) days<br>26,232 (84.2%) received PPI within 1 day of admission
Duration of PPI treatment	Median (IQR) of 6 (3, 13) days for all patients<br>Median (IQR) of 9 (4, 22) days for the lansoprazole group<br>Median (IQR) of 6 (3, 12) days for the other PPI group

IQR = interquartile range; PPI = proton-pump inhibitor

© 2023 Bai D et al. *JAMA Network Open.*

eTable 4. Excluded patients on no proton pump inhibitor and patients with missing data

	Lansoprazole (N=3,747)	Other PPI (N=27,405)	Excluded patients on ceftriaxone and no PPI (N=49,573)	Patients with missing data (N=2,895)
Age Mean (SD)	74.0 (15.9)	71.4 (16.0)	69.7 (19.0)	72.0 (18.2)
Sex				
Female	1,772 (47.3%)	13,245 (48.3%)	23,414 (47.2%)	1,411 (48.7%)
Male	1,975 (52.7%)	14,160 (51.7%)	26,159 (52.8%)	1,484 (51.3%)
From a long-term care home	574 (15.3%)	5,345 (6.9%)	3,750 (7.6%)	160 (5.5%)
Hospital sites				
A	452 (12.1%)	5,118 (18.7%)	6,346 (12.8%)	63 (2.2%)
B	0 (0%)	10 (0.04%)	356 (0.7%)	1 (0.03%)
C	439 (11.7%)	3,206 (11.7%)	4,471 (9.0%)	2,362 (81.6%)
D	431 (11.5%)	1,246 (4.6%)	3,216 (6.5%)	271 (9.4%)
E	48 (1.3%)	415 (1.5%)	835 (1.7%)	6 (0.2%)
F	223 (6.0%)	693 (2.5%)	2,768 (5.6%)	11 (0.4%)
G	8 (0.2%)	890 (3.3%)	1,035 (2.1%)	4 (0.1%)
H	116 (3.1%)	2,325 (8.5%)	4,493 (9.1%)	22 (0.8%)
I	43 (1.2%)	1,135 (4.1%)	3,759 (7.6%)	15 (0.5%)
J	562 (15.0%)	2,601 (9.5%)	5,261 (10.6%)	11 (0.4%)
K	760 (20.3%)	2,818 (10.3%)	6,186 (12.5%)	56 (1.9%)
L	338 (9.0%)	4,168 (15.2%)	5,725 (11.6%)	46 (1.6%)
M	327 (8.7%)	2,780 (10.1%)	5,122 (10.3%)	27 (0.9%)
Admission year				
2015	246 (6.6%)	2,427 (8.9%)	4,677 (9.4%)	822 (28.4%)
2016	390 (10.4%)	3,312 (12.1%)	6,505 (13.1%)	1,456 (50.3%)
2017	548 (14.6%)	4,108 (15.0%)	8,008 (16.2%)	261 (9.0%)
2018	655 (17.5%)	4,331 (15.8%)	8,423 (17.0%)	97 (3.4%)
2019	573 (15.3%)	4,613 (16.8%)	8,061 (16.3%)	121 (4.2%)
2020	923 (24.6%)	5,391 (19.7%)	8,982 (18.1%)	79 (2.7%)
2021	412 (11.0%)	3,223 (11.8%)	4,917 (9.9%)	59 (2.0%)
Admission season				
Winter	974 (26.0%)	6,887 (25.1%)	12,599 (25.4%)	764 (26.4%)
Spring	996 (26.6%)	6,837 (25.0%)	12,464 (25.1%)	655 (22.6%)
Summer	901 (24.1%)	7,005 (25.6%)	12,455 (25.1%)	713 (24.6%)
Autumn	876 (23.4%)	6,676 (24.4%)	12,055 (24.3%)	763 (26.4%)
Modified Charlson comorbidity index Mean (SD)	1.6 (1.8)	1.7 (1.9)	1.0 (1.6)	1.0 (1.7)
Risk factors for ventricular arrhythmia				
Coronary artery disease	94 (2.5%)	728 (2.7%)	932 (1.9%)	91 (3.1%)
Prior myocardial infarction	68 (1.8%)	551 (2.0%)	781 (1.6%)	89 (3.1%)
Cardiomyopathy	43 (1.2%)	251 (0.9%)	396 (0.8%)	22 (0.8%)
Heart failure	484 (12.9%)	3,407 (12.4%)	4,933 (10.0%)	327 (11.3%)
Prior ventricular arrhythmia	7 (0.2%)	69 (0.3%)	129 (0.3%)	7 (0.2%)
Chronic kidney disease	203 (5.4%)	1,748 (6.4%)	2,262 (4.6%)	271 (9.4%)
Abnormal serum potassium level at admission	1,706 (45.5%)	11,614 (42.4%)	20,264 (40.9%)	100 (3.5%)
mLAPS score Mean (SD)	27.0 (18.5)	24.5 (16.8)	22.6 (16.2)	32.2 (19.8)
ICU admission prior to ceftriaxone and PPI	349 (9.3%)	509 (1.9%)	N/A	N/A
Admitting main responsible diagnosis				
Pneumonia	251 (6.7%)	1,881 (6.9%)	3,758 (7.6%)	267 (9.2%)
Urinary tract infection	203 (5.4%)	1,642 (6.0%)	3,485 (7.0%)	182 (6.3%)
Aspiration	418 (11.2%)	718 (2.6%)	2,061 (4.2%)	89 (3.1%)
COPD with lower respiratory tract infection	109 (2.9%)	1,166 (4.3%)	1,712 (3.5%)	98 (3.4%)
Congestive heart failure	103 (2.8%)	1,030 (3.8%)	1,544 (3.1%)	82 (2.8%)
COPD exacerbation	47 (1.3%)	874 (3.2%)	1,472 (3.0%)	90 (3.1%)
Sepsis	107 (2.9%)	555 (2.0%)	1,224 (2.5%)	59 (2.0%)
COVID-19 infection	144 (3.8%)	448 (1.6%)	986 (2.0%)	8 (0.3%)

© 2023 Bai D et al. *JAMA Network Open.*

	Lansoprazole (N=3,747)	Other PPI (N=27,405)	Excluded patients on ceftriaxone and no PPI (N=49,573)	Patients with missing data (N=2,895)
Cellulitis involving limb	15 (0.4%)	293 (1.1%)	1,020 (2.1%)	76 (2.6%)
Acute renal failure	53 (1.4%)	326 (1.6%)	777 (1.6%)	47 (1.6%)
Medications taken during ceftriaxone therapy				
Quinolones	162 (4.3%)	796 (2.9%)	1,397 (2.8%)	213 (7.4%)
Macrolides	109 (2.9%)	536 (2.0%)	1,015 (2.1%)	31 (1.1%)
Cardiac medications associated with ventricular arrhythmia	226 (6.0%)	615 (2.2%)	863 (1.7%)	89 (3.1%)
Other medications associated with ventricular arrhythmia	704 (18.8%)	3,532 (12.9%)	5,200 (10.5%)	336 (11.6%)
Outcome				
Ventricular arrhythmia or cardiac arrest	126 (3.4%)	319 (1.2%)	402 (0.8%)	38 (1.3%)
In-hospital mortality	746 (19.9%)	2,762 (10.1%)	4,821 (9.7%)	309 (10.7%)

COPD = chronic obstructive pulmonary disease; ICU = intensive care unit; mLAPS = modified laboratory-based acute physiology score; N/A = not applicable; SD = standard deviation

© 2023 Bai D et al. *JAMA Network Open.*

eTable 5. Proportion of patients who were also prescribed another proton pump inhibitor during hospital stay in the lansoprazole and other proton pump inhibitor group

	Lansoprazole group (N=3,747)	Pantoprazole group (N=25,699)	Rabeprazole group (N=1,062)	Esomeprazole group (N=434)	Omeprazole group (N=210)
Lansoprazole use	3,747 (100%)	0 (0%)	0 (0%)	0 (0%)	0 (0%)
Pantoprazole use	1,662 (44.4%)	25,699 (100%)	123 (11.6%)	163 (37.6%)	40 (19.1%)
Rabeprazole use	68 (1.8%)	0 (0%)	1,062 (100%)	3 (0.7%)	6 (2.9%)
Esomeprazole use	29 (0.8%)	0 (0%)	0 (0%)	434 (100%)	2 (1.0%)
Omeprazole use	17 (0.5%)	0 (0%)	0 (0%)	0 (0%)	210 (100%)

Within the lansoprazole group who were also prescribed another proton pump inhibitor, lansoprazole accounts for median of 50% (interquartile range 50% to 67%) of the total duration of proton pump inhibitor use. In 77% cases, lansoprazole accounted for the majority of the total duration of proton pump inhibitor use.

© 2023 Bai D et al. *JAMA Network Open.*

eTable 6. Hospital sites description

Hospital site	Bed size	Description	Proton pump inhibitor distribution	Event rates
A	496	Academic	Total: 5,570<br>Lansoprazole: 452 (8.1%)<br>Esomeprazole: 15 (0.3%)<br>Omeprazole: 11 (0.2%)<br>Pantoprazole: 5,086 (91.3%)<br>Rabeprazole: 6 (0.1%)	Total: 5,570<br>Ventricular arrhythmia or cardiac arrest: 43 (0.8%)<br>In-hospital mortality: 574 (10.3%)
B	556	Community	Total: 10<br>Lansoprazole: 0 (0%)<br>Esomeprazole: 0 (0%)<br>Omeprazole: 0 (0%)<br>Pantoprazole: 10 (100%)<br>Rabeprazole: 0 (0%)	Total: 10<br>Ventricular arrhythmia or cardiac arrest: 0 (0%)<br>In-hospital mortality: 1 (10.0%)
C	457	Community	Total: 3,645<br>Lansoprazole: 439 (12.0%)<br>Esomeprazole: 12 (0.3%)<br>Omeprazole: 4 (0.1%)<br>Pantoprazole: 3,177 (87.2%)<br>Rabeprazole: 13 (0.4%)	Total: 3,645<br>Ventricular arrhythmia or cardiac arrest: 49 (1.3%)<br>In-hospital mortality: 316 (8.7%)
D	753	Academic<br>Centre of excellence for obstetrics medicine, gastroenterology, surgical oncology	Total: 1,677<br>Lansoprazole: 431 (25.7%)<br>Esomeprazole: 39 (2.3%)<br>Omeprazole: 76 (4.5%)<br>Pantoprazole: 1,046 (62.4%)<br>Rabeprazole: 85 (5.1%)	Total: 1,677<br>Ventricular arrhythmia or cardiac arrest: 35 (2.1%)<br>In-hospital mortality: 221 (13.2%)
E	354	Community	Total: 463<br>Lansoprazole: 48 (10.4%)<br>Esomeprazole: 6 (1.3%)<br>Omeprazole: 21 (4.5%)<br>Pantoprazole: 91 (19.7%)<br>Rabeprazole: 297 (64.2%)	Total: 463<br>Ventricular arrhythmia or cardiac arrest: 5 (1.1%)<br>In-hospital mortality: 74 (15.9%)
F	1,096	Academic<br>Trauma centre	Total: 916<br>Lansoprazole: 223 (24.3%)<br>Esomeprazole: 10 (1.1%)<br>Omeprazole: 46 (5.0%)<br>Pantoprazole: 566 (61.8%)	Total: 916<br>Ventricular arrhythmia or cardiac arrest: 22 (2.4%)<br>In-hospital mortality: 78 (8.5%)

© 2023 Bai D et al. *JAMA Network Open.*

			Rabeprazole: 71 (7.8%)	
Hospital site	Bed size	Description	Proton pump inhibitor distribution	Event rates
G	191	Community	Total: 898<br>Lansoprazole: 8 (0.9%)<br>Esomeprazole: 13 (1.5%)<br>Omeprazole: 1 (0.1%)<br>Pantoprazole: 874 (97.3%)<br>Rabeprazole: 2 (0.2%)	Total: 898<br>Ventricular arrhythmia or cardiac arrest: 9 (1.0%)<br>In-hospital mortality: 110 (12.3%)
H	527	Academic<br>Trauma centre<br>Centre of excellence for care of homeless and disadvantaged	Total: 2,441<br>Lansoprazole: 116 (4.8%)<br>Esomeprazole: 108 (4.4%)<br>Omeprazole: 0 (0%)<br>Pantoprazole: 2,052 (84.1%)<br>Rabeprazole: 165 (6.8%)	Total: 2,441<br>Ventricular arrhythmia or cardiac arrest: 19 (0.8%)<br>In-hospital mortality: 216 (8.9%)
I	366	Community	Total: 1,178<br>Lansoprazole: 43 (3.7%)<br>Esomeprazole: 0 (0%)<br>Omeprazole: 11 (0.9%)<br>Pantoprazole: 1,086 (92.2%)<br>Rabeprazole: 38 (3.2%)	Total: 1,178<br>Ventricular arrhythmia or cardiac arrest: 30 (2.6%)<br>In-hospital mortality: 170 (14.4%)
J	382	Community	Total: 3,163<br>Lansoprazole: 562 (17.8%)<br>Esomeprazole: 9 (0.3%)<br>Omeprazole: 15 (0.5%)<br>Pantoprazole: 2,195 (69.4%)<br>Rabeprazole: 382 (12.1%)	Total: 3,163<br>Ventricular arrhythmia or cardiac arrest: 51 (1.6%)<br>In-hospital mortality: 324 (10.2%)
K	751	Community	Total: 3,578<br>Lansoprazole: 760 (21.2%)<br>Esomeprazole: 8 (0.2%)<br>Omeprazole: 20 (0.6%)<br>Pantoprazole: 2,790 (78.0%)<br>Rabeprazole: 0 (0%)	Total: 3,578<br>Ventricular arrhythmia or cardiac arrest: 71 (2.0%)<br>In-hospital mortality: 570 (15.9%)
L	417	Academic<br>Centre of excellence for cardiology, cardiac surgery and solid organ transplant	Total: 4,506<br>Lansoprazole: 338 (7.5%)<br>Esomeprazole: 88 (2.0%)<br>Omeprazole: 5 (0.1%)<br>Pantoprazole: 4,072 (90.4%)	Total: 4,506<br>Ventricular arrhythmia or cardiac arrest: 72 (1.6%)<br>In-hospital mortality: 547 (12.1%)

© 2023 Bai D et al. *JAMA Network Open.*

			Rabeprazole: 3 (0.1%)	
Hospital site	Bed size	Description	Proton pump inhibitor distribution	Event rates
M	272	Academic<br>Centre of excellence for neurosurgery and neurology	Total: 3,107<br>Lansoprazole: 327 (10.5%)<br>Esomeprazole: 126 (4.1%)<br>Omeprazole: 0 (0%)<br>Pantoprazole: 2,654 (85.4%)<br>Rabeprazole: 0 (0%)	Total: 3,107<br>Ventricular arrhythmia or cardiac arrest: 39 (1.3%)<br>In-hospital mortality: 307 (9.9%)

© 2023 Bai D et al. *JAMA Network Open.*

eTable 7. The association of a priori selected predictor with ventricular arrhythmia or cardiac arrest in logistic regression model

Predictors	Univariate<br>Unadjusted OR (95% CI)	Multivariable<br>Adjusted OR (95% CI)
Age per year	1.00 (0.99 to 1.00)	1.00 (0.99 to 1.00)
Sex		
Female	Reference	Reference
Male	1.40 (1.16 to 1.70)	1.35 (1.11 to 1.64)
Modified Charlson comorbidity index per point	1.10 (1.05 to 1.15)	1.09 (1.04 to 1.14)
Risk factors for ventricular arrhythmias		
Coronary artery disease	1.02 (0.54 to 1.74)	1.34 (0.70 to 2.31)
Prior myocardial infarction	1.99 (1.17 to 3.14)	2.35 (1.35 to 3.81)
Cardiomyopathy	4.08 (2.35 to 6.59)	3.59 (2.00 to 5.99)
Heart failure	2.22 (1.78 to 2.76)	2.69 (2.10 to 3.42)
Prior arrhythmia	1.87 (0.31 to 5.96)	1.65 (0.26 to 5.65)
Chronic kidney disease	1.16 (0.79 to 1.64)	1.38 (0.93 to 1.98)
Abnormal serum potassium level at admission	1.59 (1.32 to 1.92)	1.34 (1.10 to 1.62)
mLAPS score	1.02 (1.01 to 1.02)	1.01 (1.01 to 1.02)
ICU admission prior to ceftriaxone and PPI	5.84 (4.37 to 7.67	2.12 (1.49 to 2.99)
Medications taken during ceftriaxone therapy		
Quinolones	1.49 (0.92 to 2.29)	0.83 (0.50 to 1.31)
Macrolides	1.32 (0.70 to 2.24)	0.62 (0.32 to 1.09)
Cardiac medications associated with ventricular arrhythmia	5.97 (4.47 to 7.85)	2.48 (1.75 to 3.48)
Other medications associated with ventricular arrhythmia	2.28 (1.83 to 2.81)	1.59 (1.24 to 2.02)
Lansoprazole	2.95 (2.39 to 3.63)	2.33 (1.86 to 2.91)

CI = confidence interval; ICU = intensive care unit; OR = odds ratio; PPI = proton pump inhibitor

© 2023 Bai D et al. *JAMA Network Open.*

eTable 8. Risk of ventricular arrhythmia or cardiac arrest, and in-hospital mortality per year from 2015 to 2021

	2015	2016	2017	2018	2019	2020	2021
Total number of patients	2,673	3,702	4,656	4,986	5,186	6,314	3,635
Ventricular arrhythmia or cardiac arrest							
Number of events	56 (2.1%)	53 (1.4%)	73 (1.6%)	70 (1.4%)	73 (1.4%)	81 (1.3%)	39 (1.1%)
Lansoprazole vs other PPI Risk ratio (95% CI)	1.6 (0.8-3.4)	1.7 (0.9-3.5)	3.0 (1.8-5.0)	3.0 (1.8-5.0)	3.3 (2.0-5.4)	4.2 (2.7-6.5)	3.1 (1.5-6.1)
In-hospital mortality							
Number of events	298 (11.2%)	419 (11.3%)	517 (11.1%)	540 (10.8%)	585 (11.3%)	708 (11.2%)	441 (12.1%)
Lansoprazole vs other PPI Risk ratio (95% CI)	1.9 (1.4-2.5)	1.8 (1.5-2.3)	1.8 (1.5-2.2)	2.1 (1.8-2.5)	2.3 (1.9-2.7)	2.1 (1.8-2.4)	1.7 (1.4-2.2)

© 2023 Bai D et al. *JAMA Network Open.*

eTable 9. Baseline characteristics within subgroup analysis of patients not admitted to ICU prior to ceftriaxone and PPI

	Lansoprazole (N=3,398)	Other PPI (N=26,896)	ASMD
Age Mean (SD)	74.7 (15.8)	71.6 (16.0)	0.198
Sex			
Female	1,633 (48.1%)	13,035 (48.5%)	0.008
Male	1,765 (51.9%)	13,861 (51.5%)	0.008
From a long-term care home	563 (16.6%)	1,570 (5.8%)	0.345
Hospital sites			
A	407 (12.0%)	5,074 (18.9%)	0.192
B	0 (0%)	10 (0.04%)	0.027
C	387 (11.4%)	3,152 (11.7%)	0.010
D	374 (11.0%)	1,153 (4.3%)	0.255
E	47 (1.4%)	415 (1.5%)	0.013
F	211 (6.2%)	681 (2.5%)	0.181
G	8 (0.2%)	865 (3.2%)	0.230
H	87 (2.6%)	2,234 (8.3%)	0.256
I	40 (1.2%)	1,106 (4.1%)	0.184
J	536 (15.8%)	2,588 (9.6%)	0.186
K	722 (21.3%)	2,791 (10.4%)	0.301
L	303 (8.9%)	4,104 (15.3%)	0.195
M	276 (8.1%)	2,723 (10.1%)	0.070
Admission year			
2015	217 (6.4%)	2,345 (8.7%)	0.088
2016	360 (10.6%)	3,245 (12.1%)	0.046
2017	514 (15.1%)	4,024 (15.0%)	0.005
2018	609 (17.9%)	4,263 (15.9%)	0.055
2019	508 (15.0%)	4,545 (16.9%)	0.053
2020	821 (24.2%)	5,310 (19.7%)	0.107
2021	369 (10.9%)	3,164 (11.8%)	0.029
Admission season			
Winter	888 (26.1%)	6,772 (25.2%)	0.022
Spring	903 (26.6%)	6,705 (24.9%)	0.038
Summer	819 (24.1%)	6,878 (25.6%)	0.034
Autumn	788 (23.2%)	6,541 (24.3%)	0.027
Modified Charlson comorbidity index Mean (SD)	1.3 (1.7)	1.4 (1.9)	0.075
Risk factors for ventricular arrhythmia			
Coronary artery disease	86 (2.5%)	713 (2.7%)	0.008
Prior myocardial infarction	59 (1.7%)	522 (1.9%)	0.015
Cardiomyopathy	33 (1.0%)	240 (0.9%)	0.008
Heart failure	438 (12.9%)	3,351 (12.5%)	0.013
Prior ventricular arrhythmia	<6 (<0.2%)	64 (0.2%)	0.029
Chronic kidney disease	178 (5.2%)	1,721 (6.4%)	0.050
Abnormal serum potassium level at admission	1,505 (44.3%)	11,317 (42.1%)	0.045
mLAPS score Mean (SD)	26.3 (17.6)	24.3 (16.5)	0.119
ICU admission prior to ceftriaxone and PPI	0 (0%)	0 (0%)	0

© 2023 Bai D et al. *JAMA Network Open.*

	Lansoprazole (N=3,398)	Other PPI (N=26,896)	ASMD
Admitting main responsible diagnosis			
Pneumonia	240 (7.1%)	1,874 (7.0%)	0.004
Urinary tract infection	200 (5.9%)	1,634 (6.1%)	0.008
Aspiration	404 (11.9%)	703 (2.6%)	0.364
COPD with lower respiratory tract infection	101 (3.0%)	1,154 (4.3%)	0.071
Congestive heart failure	96 (2.8%)	1,022 (3.8%)	0.054
COPD exacerbation	42 (1.2%)	863 (3.2%)	0.134
Sepsis	105 (3.1%)	523 (1.9%)	0.073
COVID-19 infection	114 (3.4%)	435 (1.6%)	0.112
Cellulitis involving limb	15 (0.4%)	292 (1.1%)	0.074
Acute renal failure	48 (1.4%)	420 (1.6%)	0.012
Medications taken during ceftriaxone therapy			
Quinolones	115 (3.4%)	749 (2.8%)	0.035
Macrolides	59 (1.7%)	476 (1.8%)	0.003
Cardiac medications associated with ventricular arrhythmia	87 (2.6%)	489 (1.8%)	0.051
Other medications associated with ventricular arrhythmia	517 (15.2%)	3,314 (12.3%)	0.084

Abbreviations: ASMD = absolute standardized mean difference; COPD = chronic obstructive pulmonary disease; ICU = intensive care unit; mLAPS = modified laboratory-based acute physiology score; PPI = proton pump inhibitor; SD = standard deviation

© 2023 Bai D et al. *JAMA Network Open.*

eTable 10. Outcomes within subgroup analysis of patients not admitted to ICU prior to ceftriaxone and PPI

	Lansoprazole (N=3,398)	Other PPI (N=26,896)	Unadjusted risk (95% CI) P-value[a]	Adjusted risk by overlap weight (95% CI)
Ventricular arrhythmia or cardiac arrest	90 (2.7%)	295 (1.1%)	RD in %: 1.6 (1.0 to 2.2)<br>RR: 2.4 (1.9 to 3.0)<br>P<0.0001	RD in %: 1.6 (1.0 to 2.2)<br>RR: 2.4 (1.8 to 3.0)
All cause in-hospital mortality	670 (19.7%)	2,668 (9.9%)	RD in %: 9.8 (8.4 to 11.2)<br>RR: 2.0 (1.8 to 2.1)<br>P<0.0001	RD in %: 7.8 (6.4 to 9.2)<br>RR: 1.7 (1.5 to 1.8)
Transfer to ICU	126 (3.7%)	1,192 (4.4%)	RD in %: -0.7 (-1.4 to 0.0)<br>RR: 0.8 (0.7 to 1.0)<br>P=0.0569	RD in %: -0.1 (-0.8 to 0.6)<br>RR: 1.0 (0.8 to 1.2)

[a]P-value by Chi squared test

© 2023 Bai D et al. *JAMA Network Open.*

eTable 11. Baseline characteristics within subgroup analysis of patients who received either only lansoprazole or only pantoprazole without being exposed to any other PPI during hospital stay

	Lansoprazole only (N=1,991)	Pantoprazole only (N=25,699)	ASMD
Age Mean (SD)	73.1 (16.5)	71.3 (16.1)	0.112
Sex			
Female	943 (47.4%)	12,317 (47.9%)	0.011
Male	1,048 (52.6%)	13,382 (52.1%)	0.011
From a long-term care home	303 (15.2%)	1,479 (5.8%)	0.313
Hospital sites			
A	172 (8.6%)	5,086 (19.8%)	0.324
B	0 (0%)	10 (0.04%)	0.028
C	251 (12.6%)	3,177 (12.4%)	0.007
D	297 (14.9%)	1,046 (4.1%)	0.377
E	26 (1.3%)	91 (0.4%)	0.105
F	147 (7.4%)	566 (2.2%)	0.244
G	0 (0%)	874 (3.4%)	0.265
H	76 (3.8%)	2,052 (8.0%)	0.178
I	20 (1.0%)	1,086 (4.2%)	0.203
J	217 (10.9%)	2,195 (8.5%)	0.080
K	328 (16.5%)	2,790 (10.9%)	0.164
L	227 (11.4%)	4,072 (15.8%)	0.130
M	230 (11.6%)	2,654 (10.3%)	0.039
Admission year			
2015	128 (6.4%)	2,192 (8.5%)	0.080
2016	183 (9.2%)	3,018 (11.7%)	0.083
2017	282 (14.2%)	3,844 (15.0%)	0.023
2018	326 (16.4%)	4,123 (16.0%)	0.009
2019	311 (15.6%)	4,428 (17.2%)	0.043
2020	518 (26.0%)	5,081 (19.8%)	0.149
2021	243 (12.2%)	3,013 (11.7%)	0.015
Admission season			
Winter	506 (25.4%)	6,436 (25.0%)	0.009
Spring	546 (27.4%)	6,414 (25.0%)	0.056
Summer	482 (24.2%)	6,582 (25.6%)	0.032
Autumn	457 (23.0%)	6,267 (24.4%)	0.034
Modified Charlson comorbidity index Mean (SD)	1.1 (1.6)	1.4 (1.9)	0.147
Risk factors for ventricular arrhythmia			
Coronary artery disease	49 (2.5%)	685 (2.7%)	0.013
Prior myocardial infarction	34 (1.7%)	526 (2.1%)	0.025
Cardiomyopathy	23 (1.2%)	235 (0.9%)	0.024
Heart failure	242 (12.2%)	3,192 (12.4%)	0.008
Prior ventricular arrhythmia	6 (0.3%)	66 (0.3%)	0.008
Chronic kidney disease	101 (5.1%)	1,666 (6.5%)	0.060
Abnormal serum potassium level at admission	893 (44.9%)	10,865 (42.3%)	0.052
mLAPS score Mean (SD)	25.8 (18.1)	24.5 (16.8)	0.074
ICU admission prior to ceftriaxone and PPI	214 (10.8%)	475 (1.9%)	0.373

© 2023 Bai D et al. *JAMA Network Open.*

	Lansoprazole only (N=1,991)	Pantoprazole only (N=25,699)	ASMD
Admitting main responsible diagnosis			
Pneumonia	139 (7.0%)	1,734 (6.8%)	0.009
Urinary tract infection	111 (5.6%)	1,479 (5.8%)	0.008
Aspiration	243 (12.2%)	648 (2.5%)	0.377
COPD with lower respiratory tract infection	56 (2.8%)	1,063 (4.1%)	0.072
Congestive heart failure	47 (2.4%)	967 (3.8%)	0.081
COPD exacerbation	22 (1.1%)	832 (3.2%)	0.147
Sepsis	50 (2.5%)	516 (2.0%)	0.034
COVID-19 infection	89 (4.5%)	437 (1.7%)	0.161
Cellulitis involving limb	11 (0.6%)	271 (1.1%)	0.056
Acute renal failure	27 (1.4%)	403 (1.6%)	0.018
Medications taken during ceftriaxone therapy			
Quinolones	104 (5.2%)	754 (2.9%)	0.116
Macrolides	69 (3.5%)	505 (2.0%)	0.092
Cardiac medications associated with ventricular arrhythmia	142 (7.1%)	583 (2.3%)	0.231
Other medications associated with ventricular arrhythmia	419 (21.0%)	3,359 (13.1%)	0.213

Abbreviations: ASMD = absolute standardized mean difference; COPD = chronic obstructive pulmonary disease; ICU = intensive care unit; mLAPS = modified laboratory-based acute physiology score; PPI = proton pump inhibitor; SD = standard deviation

© 2023 Bai D et al. *JAMA Network Open.*

eTable 12. Outcomes within subgroup analysis of patients who received either only lansoprazole or only pantoprazole without being exposed to any other PPI during hospital stay

	Lansoprazole only (N=1,991)	Pantoprazole only (N=25,699)	Unadjusted risk (95% CI) P-value[a]	Adjusted risk by overlap weight (95% CI)
Ventricular arrhythmia or cardiac arrest	55 (2.8%)	296 (1.2%)	RD in %: 1.6 (1.0 to 2.4)<br>RR: 2.4 (1.8 to 3.2)<br>P<0.0001	RD in %: 1.1 (0.3 to 1.8)<br>RR: 1.7 (1.2 to 2.3)
All cause in-hospital mortality	342 (17.2%)	2,609 (10.2%)	RD in %: 7.0 (5.4 to 8.8)<br>RR: 1.7 (1.5 to 1.9)<br>P<0.0001	RD in %: 4.8 (3.0 to 6.5)<br>RR: 1.4 (1.2 to 1.6)

[a]P-value by Chi squared test
CI = confidence interval; RD = risk difference; RR = risk ratio

© 2023 Bai D et al. *JAMA Network Open.*

eTable 13. Sensitivity analysis of outcomes during hospital stay that includes patients with missing mLAPS and assumes best-worst-case scenario for unknown exposure[a]

Outcome	No. (%)		Unadjusted risk (95% CI)<br>P-value
	Lansoprazole (N=4,135)	Other PPI (N=28,389)	
Ventricular arrhythmia or cardiac arrest	131 (3.2)	332 (1.2)	RD in %: 2.0 (1.5 to 2.6)<br>RR: 2.7 (2.2 to 3.3)<br>P<0.001
All cause in-hospital mortality	782 (18.9)	2,870 (10.1)	RD in %: 8.8 (7.6 to 10.1)<br>RR: 1.9 (1.7 to 2.0)<br>P<0.001

[a]The best-worst-case scenario assigned all patients with unknown exposure and an event (ventricular arrhythmia, cardiac arrest, and/or in-hospital death) to the other PPI group, whereas all patients with unknown exposure and no event were assigned to the lansoprazole group

CI = confidence interval; PPI = proton pump inhibitor; RD = risk difference; RR = risk ratio

© 2023 Bai D et al. *JAMA Network Open.*

eTable 14. Other sensitivity analyses

Sensitivity analysis	Outcome	Events	Unadjusted risk (95% CI)	Adjusted risk by overlap weights (95% CI)
Adjusting for ceftriaxone and PPI duration in propensity score	Ventricular arrhythmia or cardiac arrest	Lansoprazole: 126/3,747 (3.4%) Other PPI: 319/27,405 (1.2%)	RD in %: 2.2 (1.7 to 2.8) RR: 2.9 (2.4 to 3.5)	RD in %: 1.7 (1.1 to 2.2) RR: 2.1 (1.7 to 2.7)
	All cause in-hospital mortality	Lansoprazole: 746/3,747 (19.9%) Other PPI: 2,762/27,405 (10.1%)	RD in %: 9.8 (8.5 to 11.2) RR: 2.0 (1.8 to 2.1)	RD in %: 7.4 (6.0 to 8.7) RR: 1.6 (1.5 to 1.7)
Including only patients who had ceftriaxone and PPI started within 1 day of admission	Ventricular arrhythmia or cardiac arrest	Lansoprazole: 26/1,720 (1.5%) Other PPI: 180/21,528 (0.8%)	RD in %: 0.7 (0.2 to 1.4) RR: 1.8 (1.2 to 2.7)	RD in %: 0.6 (0 to 1.3) RR: 1.7 (1.1 to 2.6)
	All cause in-hospital mortality	Lansoprazole: 197/1,720 (11.5%) Other PPI: 1,721/21,528 (8.0%)	RD in %: 3.5 (2.0 to 5.1) RR: 1.4 (1.2 to 1.6)	RD in %: 2.3 (0.7 to 3.9) RR: 1.3 (1.1 to 1.4)
Subgroup analysis including only PPIs that are administered orally	Ventricular arrhythmia or cardiac arrest	Lansoprazole: 126/3,747 (3.4%) Other PPI: 23/1,706 (1.4%)	RD in %: 2.0 (1.2 to 2.8) RR: 2.5 (1.6 to 3.9)	RD in %: 1.2 (0.2 to 2.3) RR: 1.3 (1.1 to 3.0)
	All cause in-hospital mortality	Lansoprazole: 746/3,747 (19.9%) Other PPI: 153/1,706 (9.0%)	RD in %: 10.9 (9.0 to 12.8) RR: 2.2 (1.9 to 2.6)	RD in %: 8.5 (6.1 to 10.9) RR: 1.9 (1.6 to 2.4)

CI = confidence interval; PPI = proton pump inhibitor; RD = risk difference; RR = risk ratio

© 2023 Bai D et al. *JAMA Network Open.*
