# Supplementary material for: Ceftriaxone and the Risk of Ventricular Arrhythmia, Cardiac Arrest, and Death Among Patients Receiving Lansoprazole
Source: JAMA Netw Open. 2023 Oct 26;6(10):e2339893. doi: 10.1001/jamanetworkopen.2023.39893 (PMC10603497; doi:10.1001/jamanetworkopen.2023.39893)
Supplement: Supplement 2. — Data Sharing Statement [file jamanetwopen-e2339893-s002.pdf]

Data Sharing Statement

Bai. Ceftriaxone and the Risk of Ventricular Arrhythmia, Cardiac Arrest, and Death Among Patients Receiving Lansoprazole. *JAMA Netw Open*. Published October 26, 2023. doi:10.1001/jamanetworkopen.2023.39893

Data
Data available: No
